# Navigating the Intersection of PrEP and Medicaid

**DOI:** 10.1017/jme.2022.38

**Published:** 2022

**Authors:** Naomi Seiler, Claire Heyison, Gregory Dwyer, Aaron Karacuschansky, Paige Organick-Lee, Alexis Osei, Helen Stoll, Katie Horton

**Keywords:** PrEP, HIV, Medicaid

## Abstract

The proposed national PrEP program would serve people who are uninsured as well as those enrolled in Medicaid. In this article, the authors propose a set of recommendations for the proposed program’s implementers as well as state Medicaid agencies and Medicaid managed care organizations to ensure PrEP access for people enrolled in Medicaid, addressing gaps without undermining the important role of the Medicaid program in covering and promoting PrEP.

The proposal by Killelea and colleagues to expand PrEP services through a “Vaccines for Children” model gets to a core challenge in HIV prevention: we have a highly effective tool that is only benefitting a small, disproportionately white and wealthy fraction of potential users. If FDA had approved an HIV vaccine in 2012, it would be considered an outrage if it still were not widely available and reaching all communities. Killelea and colleagues’ proposed approach would engage new providers of PrEP services and, importantly, bring new energy and visibility into the effort to make PrEP accessible to people who choose to use it to prevent HIV acquisition.^1^

Yet, one component of the proposal raises particular questions: the extension of eligibility to people with Medicaid. Including people who are *un*insured is clearly appropriate: numerous studies have demonstrated that a lack of insurance is a major barrier to PrEP initiation.^2^ While people who are uninsured could receive PrEP medication from the manufacturer, these assistance programs do not include generics, and likely do not cover the ancillary clinical services that PrEP users need, such as office visits and HIV and STI tests.

But why include people with Medicaid in the scope of this proposed program? Killelea and colleagues make a convincing case that uptake among Medicaid enrollees has been suboptimal, and that churn for this population could lead to inconsistent PrEP access. In addition, many potential users may not be able to see Medicaid-participating PrEP providers who are culturally competent or otherwise accessible. But if the proposal is implemented with the inclusion of Medicaid enrollees, the program’s leaders and Medicaid officials should take a number of key steps:

## 1. Ensure that state Medicaid programs and Medicaid managed care organizations (MCOs) take responsibility for promoting access to PrEP through reimbursement and provider networks and by eliminating unnecessary barriers

All fifty states’ Medicaid programs cover PrEP medication, as well as the provider visits needed for PrEP counseling, initiation, and ongoing care. However, PrEP uptake within the Medicaid program continues to lag behind those who are privately insured.^3^ The creation of a program to support PrEP should not absolve state Medicaid agencies and Medicaid managed care organizations of their responsibility to reimburse PrEP medication and services. Furthermore, Medicaid agencies and MCOs can be well situated to engage providers and enrollees to promote the uptake of PrEP within the Medicaid program. Particularly with so many generic versions of Truvada now available on the market, Medicaid agencies and MCOs should see the expansion of PrEP uptake among their enrollees as a core responsibility.

Through initiatives such as these, Medicaid agencies and MCOs can complement the efforts of a national PrEP program by addressing barriers to PrEP uptake beyond cost and insurance coverage, such as gaps in provider knowledge, inconsistent or subjective PrEP screening, and lack of cultural competence in treating priority populations. In light of the continued unmet need for PrEP as well as the evolving set of available medications, the Centers for Medicare and Medicaid Services (CMS) should consider developing detailed guidance to states and MCOs to encourage implementation of these approaches.

Medicaid programs can promote access to PrEP by improving overall access to care through telehealth policy (including coverage of both video and audio services), targeted case management, and network adequacy standards that include PrEP prescribers.^4^ States that administer Medicaid through a managed care system can further incentivize PrEP screening and prescribing by writing PrEP standards into MCO contracts, and/or by designing value-based care arrangements that reward providers for meeting certain performance measures.^5^ For example, an MCO could offer incentives to providers whose patients using PrEP are receiving HIV and STI screenings at the recommended intervals. Through initiatives such as these, Medicaid agencies and MCOs can complement the efforts of a national PrEP program by addressing barriers to PrEP uptake beyond cost and insurance coverage, such as gaps in provider knowledge, inconsistent or subjective PrEP screening, and lack of cultural competence in treating priority populations.^6^ In light of the continued unmet need for PrEP as well as the evolving set of available medications, the Centers for Medicare and Medicaid Services (CMS) should consider developing detailed guidance to states and MCOs to encourage implementation of these approaches.

## 2. Establish Clear Monitoring

Like any public health initiative, the effectiveness of the PrEP proposal should be closely monitored. It is particularly important to quantify the extent to which Medicaid enrollees are using the program to ensure that Medicaid and the new program are working together effectively and without duplication.

Collection of demographic data should be conducted in a manner that encourages disclosure of pertinent information and does not present a barrier to participation. The approach could be modeled off the Ryan White Client-Level dataset, which includes age, race and ethnicity, gender, sexual orientation, income as a percent of the federal poverty level, health care coverage status, and housing status.^7^ Having program data that includes social determinants affecting HIV vulnerability, such as housing status, will be important to understanding the needs of members and creating targeted interventions. Additionally, collecting demographic data will provide a clearer understanding of the program’s impact on racial and gender disparities in PrEP uptake.^8^

## 3. Support Coordination of Care

It is not unprecedented for people to seek services from entities other than their usual source of care, for reasons including privacy and convenience. As Killelea and colleagues note, many people who are candidates for PrEP do not even have a usual source of care. However, even patients with a usual source of care — as is the case for most Medicaid enrollees^9^ — may choose to seek PrEP at a different site.^10^ A national PrEP program should determine whether and how PrEP providers should communicate with clients’ usual source of care (contingent on client permission), to ensure that all members of a patient’s care team are aware of the patient’s initiation of PrEP, acknowledging that such coordination may not be feasible or necessary in all circumstances. The program should also support providers in helping clients without a usual source of care, whether enrolled in Medicaid or uninsured, identify a provider for healthcare needs beyond PrEP.

## 4. Develop Clear Guidelines around Privacy

Currently, Medicaid enrollees may not wish to use their insurance for PrEP services for privacy reasons. Concerned individuals may include minor adolescents and young adults, including LGBTQ youth, with concerns about family members seeing explanation of benefits (EOBs) or denial notices.^11^ This concern may be shared by adults who do not want their partners or other household members to see written documentation of PrEP coverage. Reasons for concern can include stigma, not being “out” to family members or housemates, safety fears, or other factors.

These privacy concerns can and should be addressed through broader policy efforts to limit the use of “sensitive” information mailed to individuals indicated for PrEP, for both Medicaid and private insurance. For example, Illinois and New York have developed legislation and policy guidance to protect against disclosure of information related to sexual health, substance use, and/or other sensitive health areas in health plans’ explanations of benefits and billing documents.^12^ Six states allow dependent beneficiaries to request confidential insurance communications.^13^ CMS could consider issuing guidance to support other jurisdictions in considering adopting such policies to facilitate utilization of PrEP and other stigmatized or sensitive services.

In addition, if implemented, the proposed national PrEP program should take two key steps regarding privacy. First, the program should adopt privacy measures such as requiring providers to utilize patient’s preferred communication methods to help reduce the potential accidental disclosure of PrEP use.^14^

In addition, implementers of the proposed PrEP program should create clear guidelines noting that privacy concerns are a legitimate reason to allow a Medicaid enrollee to access the PrEP program for some or all PrEP services. This clarification of the “payer of last resort” policy could specify that when privacy concerns are a barrier to a client using Medicaid to cover PrEP, the new program would in fact be considered a “last resort.”

## 5. Develop Clear Guidelines around Provider Availability and Program Eligibility

A person enrolled in Medicaid may in theory have access to PrEP, but lack access to an appropriate prescriber, either because of distance, language barriers, LGBTQ competency, or other factors. As discussed above with regard to privacy concerns, the program should set clear parameters explaining when such Medicaid enrollees would be considered eligible for the PrEP program.

## Conclusion

Aligning a new national PrEP program with Medicaid will take thoughtful attention to issues of care coordination, privacy, and provider access. With these measures in place, including people with Medicaid in a national PrEP program would be an important “belt and suspenders” approach to promoting increased access to PrEP for low-income people nationwide.

## Note

The authors do not have any conflicts of interest to disclose.
